# In vitro virucidal activity of Echinaforce®, an *Echinacea purpurea* preparation, against coronaviruses, including common cold coronavirus 229E and SARS-CoV-2

**DOI:** 10.1186/s12985-020-01401-2

**Published:** 2020-09-21

**Authors:** Johanna Signer, Hulda R. Jonsdottir, Werner C. Albrich, Marc Strasser, Roland Züst, Sarah Ryter, Rahel Ackermann-Gäumann, Nicole Lenz, Denise Siegrist, Andreas Suter, Roland Schoop, Olivier B. Engler

**Affiliations:** 1 SPIEZ LABORATORY, Austrasse, 3700 Spiez, Switzerland; 2 Division of Infectious Diseases and Hospital Epidemiology, Kantonsspital St. Gallen, St. Gallen, Switzerland; 3 A.Vogel AG, Roggwil, Switzerland

**Keywords:** Antivirals, Echinacea, Coronavirus, Prevention, HCoV-229E, Common cold, SARS-CoV-1, MERS-CoV, SARS-CoV-2

## Abstract

**Background:**

Coronaviruses (CoVs) were long thought to only cause mild respiratory and gastrointestinal symptoms in humans but outbreaks of Middle East Respiratory Syndrome (MERS)-CoV, Severe Acute Respiratory Syndrome (SARS)-CoV-1, and the recently identified SARS-CoV-2 have cemented their zoonotic potential and their capacity to cause serious morbidity and mortality, with case fatality rates ranging from 4 to 35%. Currently, no specific prophylaxis or treatment is available for CoV infections. Therefore we investigated the virucidal and antiviral potential of *Echinacea purpurea* (Echinaforce®) against human coronavirus (HCoV) 229E, highly pathogenic MERS- and SARS-CoVs, as well as the newly identified SARS-CoV-2, in vitro.

**Methods:**

To evaluate the antiviral potential of the extract, we pre-treated virus particles and cells and evaluated remaining infectivity by limited dilution. Furthermore, we exposed cells to the extract after infection to further evaluate its potential as a prophylaxis and treatment against coronaviruses. We also determined the protective effect of Echinaforce® in re-constituted nasal epithelium.

**Results:**

In the current study, we found that HCoV-229E was irreversibly inactivated when exposed to Echinaforce® at 3.2 μg/ml IC_50_. Pre-treatment of cell lines, however, did not inhibit infection with HCoV-229E and post-infection treatment had only a marginal effect on virus propagation at 50 μg/ml. However, we did observe a protective effect in an organotypic respiratory cell culture system by exposing pre-treated respiratory epithelium to droplets of HCoV-229E, imitating a natural infection. The observed virucidal activity of Echinaforce® was not restricted to common cold coronaviruses, as both SARS-CoV-1 and MERS-CoVs were inactivated at comparable concentrations. Finally, the causative agent of COVID-19, SARS-CoV-2 was also inactivated upon treatment with 50μg/ml Echinaforce®.

**Conclusions:**

These results show that Echinaforce® is virucidal against HCoV-229E, upon direct contact and in an organotypic cell culture model. Furthermore, MERS-CoV and both SARS-CoV-1 and SARS-CoV-2 were inactivated at similar concentrations of the extract. Therefore we hypothesize that *Echinacea purpurea* preparations, such as Echinaforce®, could be effective as prophylactic treatment for all CoVs due to their structural similarities.

## Background

Coronaviruses (CoVs) are enveloped, positive-sense, single-stranded RNA viruses with a large genome, typically 26–32 kb in length. They belong to the family *Coronaviridae* and are capable of infecting a wide variety of hosts [1]. The CoVs capable of causing disease in humans (HCoVs) were traditionally thought to cause only mild gastrointestinal and respiratory tract symptoms. Currently, seven HCoVs have been identified. Four of those, HCoV-229E, HCoV-OC43, HCoV-NL63 and HCoV-HKU1, are non-zoonotic and cause worldwide outbreaks of upper respiratory tract infections (URTI) predominantly in the winter period [2]. These commonly circulating viruses have been thought to be responsible for 10–15% of all URTIs in humans. They replicate in the nasopharynx and generally cause mild, self-limited URTIs with short incubation periods, although lower tract respiratory infections and pneumonia have occasionally been described [3–6]. Until the emergence of Severe Acute Respiratory Syndrome (SARS)-CoV-1 in 2002, HCoVs were thought to mainly be responsible for the common cold. However, the more virulent coronaviruses, Middle East respiratory syndrome (MERS)-CoV and SARS-CoV-1 have animal reservoirs with proposed origins in bats [7] and can cause severe pneumonias with longer incubation periods and often fatal outcome [8]. SARS-CoV-1 was introduced into the human species in 2002 causing a worldwide pandemic, culminating in 8422 infections and 916 deaths [9]. MERS-CoV is endemic in dromedary camels and leads to lower respiratory tract infections in humans with a current case-fatality rate of 35.5% [10]. As of late 2019, a pneumonia outbreak caused by a novel CoV, designated SARS-CoV-2, supposedly originating from a live seafood market in Wuhan, China, has resulted in a global pandemic with almost 25 million infections and over 800.000 deaths (WHO situation report, August 31st 2020 and [11]). To date, there is a lack of established and clinically tested antiviral compounds against coronaviruses in general and, more distressingly, the zoonotic betacoronaviruses [12]. Given their increasing incidence and burden, finding an inexpensive, accessible and effective treatment for HCoVs is of utmost importance.

*Echinacea* plants have traditionally been used in North America for the prevention and treatment of cold and flu symptoms and are now one of the most widely used medical plants in both North America and Europe [13]. Several different products are on the market, not only varying in the *Echinacea* species and the parts of the plant used but also in manufacturing procedures, which, unfortunately, results in a large variability in quality and activity [14, 15]. Echinaforce® is a standardized preparation extracted from freshly harvested *Echinacae purpurea* plants with a 65% alcoholic solution.

Echinaforce® as prevention and treatment of respiratory tract infections has been investigated in both pre-clinical and clinical studies and its beneficial effects documented [16–19]. Specific mechanism of action is not fully understood but in vitro studies indicate that Echinaforce® inhibits enveloped respiratory viruses including influenza A and B, respiratory syncytial virus (RSV) or parainfluenza virus, through direct interaction with whole virions and viral envelope proteins [20, 21]. In general, intracellular activity of *Echinacea* has been observed for some viruses (e.g. influenza and herpes simplex virus) but not others (e.g. RSV), and only at higher concentrations than required for extracellular inactivation. Furthermore, *Echinacea* has been shown to interfere with virus mediated cytokine release [22, 23] and since typical symptoms of the common cold, i.e. sneezing, coughing and runny nose, are the results of the stimulation of pro-inflammatory cytokines, the reduction of cytokine release might help to ease such symptoms. In a randomized, double-blind, multi-center, non-inferiority clinical trial Echinaforce® was demonstrated to be non-inferior to Oseltamivir in patients with influenza-like illness, i.e. involvement of the lower respiratory tract (cough) and systemic symptoms (e.g. headache, myalgia, fever), and confirmed influenza infection with a non-significant trend towards lower incidence of complications with Echinaforce Hot Drink® compared to Oseltamivir [17].

The antiviral activity of *Echinacea* has been investigated in vitro for most of the respiratory viruses associated with common colds and flu, but as of yet, not for coronaviruses. Since HCoV-229E is a typical representative of a coronavirus strain causing a seasonal common cold, we used this virus strain to investigate the general antiviral activity of Echinaforce® against coronaviruses, thereby closing the knowledge gap on the antiviral effects of *Echinacea purpurea* on typical common cold viruses. Furthermore, we expanded our analysis to other coronaviruses, i.e. MERS-CoV, SARS-CoV-1 and SARS-CoV-2. Additionally, we utilized an organotypic respiratory cell culture system (MucilAir™) of nasal origin to investigate the protective effect of Echinaforce® against HCoV-229E in a culture system that closely mimics in vivo human airway epithelium. In the current study, we observed an irreversible reduction of the infectivity of four coronaviruses upon direct contact with the extract. Furthermore, a protective effect was observed upon apical pre-treatment in an organotypic airway model.

Additionally, to further test the general virucidal activity of Echinaforce® we treated viruses from other families, both enveloped and non-enveloped, with either RNA or DNA genomes. Due to the observed susceptibility of different CoVs, we expected other enveloped viruses to be inactivated by the extract as well. However, since non-enveloped viruses are usually more robust and Echinaforce® likely exerts its virucidal activity on the viral membrane directly, we expected such viruses to be more refractory to treatment. Interestingly, while Yellow fever virus, an enveloped RNA virus, was readily inactivated by Echinaforce®, Vaccinia virus, an enveloped DNA virus, was not.

## Methods

### Echinacea preparation

Echinaforce® (A.Vogel AG, Roggwil, Switzerland – hereafter referred to as Echinaforce) is derived from hydroethanolic extraction (65% v/v ethanol) of freshly harvested *Echinacea purpurea* using Good Manufacturing Practices (GMP). Echinacea herb and roots are extracted separately with 65% ethanol using a drug-to-extract ratio, DER, 1:11 and 1:12. Subsequently, the two fractions are combined at a final ratio of 95:5. The composition of typical marker compounds in the tested batch 1,023,117 is provided in Table 1. The final concentration of ethanol in the extract was 65% v/v with 16 mg/ml dry mass *Echinacea*. Experiments were performed with a standardized liquid formulation acquired directly from A. Vogel AG. This same formulation is available commercially.

**Table 1:** Pharmacologically active substances in Echinaforce (batch: 1023117). Data is presented as the mean of four independent determinations

Compound	Concentration (μg/ml)
**Caffeic acid**	0 ± 0
**Caftaric acid**	264.4 ± 13.0
**Chlorogenic acid**	40.2 ± 2.0
**Cichoric acid**	313.8 ± 0
**Cynarin**	0 ± 0
**Echinacoside**	6.9 ± 0.4
**PID 8/9**^a^	41.4± 0.2

^a^dodeca2E,4E,8Z, 10E/Z tetraenoic acid-isobutylamide

### Cell lines and viruses

Cell lines and viruses used in the current study are summarized in Tables 2 and 3, respectively.

**Table 2:** Overview of cell lines used in the current study

Name	Animal	Tissue	Medium^a^	Procured from
**Huh-7**	Human	Liver	DMEM + 10% FBS, 2 mM Glutamine, non-essential amino acids, Pen/strep, HEPES (Biochrom, Germany)	Prof. Volker Thiel, University of Bern, Switzerland
**Vero (CRL 81 TM)**	African Green Monkey	Kidney	MEM + 10% FBS, 2 mM Glutamine, non-essential amino acids, Pen/strep, HEPES (Biochrom, Germany)	ATCC (Manassas, VA, 20110 USA)
**Vero E6 (C1008)**	African Green Monkey	Kidney	MEM + 10% FBS, 2 mM Glutamine, non-essential amino acids, Pen/strep, HEPES (Biochrom, Germany)	ATCC (Manassas, VA, 20110 USA)
**A9 (85011426)**	Mouse	Areolar adipose tissue	DMEM + 10% FBS, 2 mM Glutamine, non-essential amino acids, Pen/strep, HEPES (Biochrom, Germany)	ECACC (Public Health England, Salisbury, UK)

All cells were cultured at 37 °C without CO_2_

^a^Dulbecco’s Modified Eagle Medium (DMEM), Minimum Essential Medium (MEM), Fetal Bovine Serum (FBS), Penicillin/Streptomycin (Pen/Strep, 100 U/mL)

**Table 3:** Overview of viruses used in the current study

Name	Strain	Propagated in	Medium^a^	Procured from
**HCoV**	229E	Huh-7, 33 °C	DMEM+ 5%FBS, 2 mM Glutamine, non-essential amino acids, Pen/strep, HEPES (Biochrom, Germany)	Prof. Volker Thiel, University of Bern, Switzerland [24, 25]
**MERS-CoV**	EMC	Vero, 37 °C	DMEM+ 2%FBS, 2 mM Glutamine, non-essential amino acids, Pen/strep, HEPES (Biochrom, Germany)	Prof. Volker Thiel, University of Bern, Switzerland [24, 25]
**SARS-CoV**	Frankfurt-1	Vero, 37 °C	DMEM+ 2%FBS, 2 mM Glutamine, non-essential amino acids, Pen/strep, HEPES (Biochrom, Germany)	Prof. Volker Thiel, University of Bern, Switzerland [24, 25]
**SARS-CoV-2**	BetaCoV/France/IDF0372/2020	Vero E6, 37 °C	DMEM+ 2%FBS, 2 mM Glutamine, non-essential amino acids, Pen/strep, HEPES (Biochrom, Germany)	Institute Pasteur, Paris, France via EVAg, European Virus Archive.
**Mouse parvovirus**	MVM Prototype, ATCC-1346	A9, 37 °C	DMEM+ 2%FBS, 2 mM Glutamine, non-essential amino acids, Pen/strep, HEPES (Biochrom, Germany)	The National Collection of Pathogenic Viruses, UK
**Yellow Fever virus**	17D, NCPV-0507	Vero, 37 °C	DMEM+ 2%FBS, 2 mM Glutamine, non-essential amino acids, Pen/strep, HEPES (Biochrom, Germany)	The National Collection of Pathogenic Viruses, UK
**Vaccinia virus**	Elstree (Lister Vaccine), ATCC-VR-1549	Vero, 37 °C	DMEM+ 2%FBS, 2 mM Glutamine, non-essential amino acids, Pen/strep, HEPES (Biochrom, Germany)	The National Collection of Pathogenic Viruses, UK

All viruses were cultured without CO2 in non-vented flasks, 24 well-, or 96 well-plates covered with sealing foil (Biorad, microseal B-film, MSB 1001) for the duration of experiments

^a^Dulbecco’s Modified Eagle Medium (DMEM), Minimum Essential Medium (MEM), Fetal Bovine Serum (FBS), Penicillin/Streptomycin (Pen/Strep, 100 U/mL)

### In vitro reconstituted human airway epithelia (MucilAir™)

Reconstituted human airway epithelia (MucilAir™) from nasal epithelial cells were purchased from Epithelix Sàrl, Geneva, Switzerland. Cells from three different healthy donors were used in all experiments to account for donor variability and experiments were conducted four times, in duplicates. During maintenance, basal culture medium (MucilAir™, 500 μl/24-well) was exchanged every 2–3 days while the apical side was washed gently (2–4 times) with 200 μl of media to remove residual mucus [26].

### Cell toxicity

Cell toxicity was determined by exposing Huh-7, Vero and Vero E6 cells to serial dilutions of Echinaforce and measuring cell viability by MTT assay (Vybrant® MTT Cell Proliferation Assay Kit, ThermoFisher, Rheinach, Switzerland) or Alamar Blue™ (Thermo Fisher, Reinach, Switzerland) according to the manufacturer’s protocol. For MTT assay, Echinaforce was diluted in corresponding cell culture medium to 100, 50, 20, 10, 1 and 0 μg/ml and added to 80% confluent Huh-7 or Vero cells in 96 well plates (200 μl/well). Cells were covered with sealing foil and incubated at 33 °C for 5 or 7 days, for Huh-7 and Vero cells, respectively. For analysis, fresh cell culture medium was added (200 μl/well), 10 μl of MTT stock solution added per well and cells incubated for 4 h at 37 °C. Following the incubation, 100 μl of 10%SDS in 0.01 M HCl solution (Merck Millipore, Molsheim, France) was added per well and incubated for 18 h at 37 °C. Absorbance was read in a photometer (SpectraMax Plus, Bucher Biotec, Basel, Switzerland) at 570 nm. For Alamar Blue, Echinaforce was diluted in corresponding cell culture medium to 100, 50, 20, 10, 1 and 0 μg/ml and added to 80% confluent Vero E6 cells in 24 well plates (500 μl/well) and incubated for 24 h at 37 °C. 10% v/v Alamar Blue™ was added to the cell culture medium and incubated for another 24 h. Absorbance was then read in a GloMax™ plate reader (Promega, Dübendorf, Switzerland) at 570 nm with a reference wavelength of 600 nm.

### Virucidal and antiviral activity against HCoV-229E in cell cultures

#### Pre-treatment of virus particles

4 × 10^4^ TCID_50_/ml HCoV-229E were incubated with Echinaforce diluted to 0, 2, 5, 10, 20, 40, 500 and 100 μg/ml in 2%-FBS-DMEM and incubated for 1 h at room temperature (RT) on a rocking platform. To estimate residual infectivity, treated virus dilutions were washed four times with 15–17 ml wash buffer (1:100 PBS, pH 7.4, in dH_2_O, Biochrom, Germany) and filtered through Vivaspin® 20 Ultrafiltration Units (Sartorius AG, Goettingen Germany) at 800 g for 15 min. Viruses were recovered from the Ultrafiltration Unit with glycine buffer (3750 mg/l glycine, 10 g/l beef extract, 14.6 g/l NaCl, pH 9.5, Sigma-Aldrich, Germany), and diluted in 1:10 in 5%-FBS -DMEM. Residual virus infectivity was determined by a limiting dilution assay (TCID_50_) according to Spearman-Karber [27].

#### Pre-treatment of cells

Huh-7 cells were incubated with 0, 1, 10 or 50 μg/ml *Echinaforce* in cell culture medium for 3 days at 33 °C. Thereafter, Echinaforce-containing medium was removed and cells infected with 100 TCID_50_ HCoV-229E (MOI of 0.005) for 1 h at 33 °*C*. Medium was replaced and cells further incubated for 48 h at 33 °C and virus titer in supernatant determined by limiting dilution assay.

#### Post-infection-treatment of cells

Huh-7 cells were infected with 100 TCID_50_ HCoV-229E (MOI of 0.005) for 1 h at 33 °C and after washing the cells twice with complete culture medium; medium containing 0, 1, 10 or 50 μg/ml Echinaforce was added. Cells were incubated at 33 °C for 72 h and virus titer in supernatant determined at 24 and 72 h post infection by limiting dilution assay.

### Virucidal and antiviral activity against HCoV-229E on re-differentiated respiratory epithelium

Prior to treatment, the mucus layer was removed from the apical surface of MucilAir™ respiratory cultures (Epithelix Sàrl, Geneva, Switzerland) by washing it three times with 200 μl Hank’s Balanced Salt Solution (HBSS, Cat N° 14,175,095, Thermo Fisher Scientific, Rheinach, Switzerland). Thereafter, the epithelium was pre-treated apically by incubating the inserts with 100 μl MucilAir™ culture medium containing 1, 10, or 50 μg/ml *Echinaforce* for 1 h at 33 °C, before removing the media and re-establishing air-liquid interface. The following day, 50 μl HBSS buffer containing 1, 10, or 50 μg/ml Echinaforce was added to the apical surface, followed by another 50 μl of HBSS containing 100 TCID_50_ HCoV-229E, added dropwise, and incubated for 1 h at 33 °C. Subsequently, air-liquid interface was re-established and cultures further incubated at 33 °C. Progeny virus was collected from the apical side by washing inserts with 200 μl HBSS at 24, 48, and 72 h post infection (hpi). Virus titers in apical wash were determined by limiting dilution assay.

### Virucidal activity against MERS-CoV, SARS-CoV-1, SARS-CoV-2, YFV, VACV and MVM

To evaluate the virucidal activity of Echinaforce against other viruses, we incubated 1 ml of MERS-CoV (5 × 10^4^ PFU/ml), SARS-CoV-1 (2 × 10^5^ PFU/ml), YFV (4 × 10^5^ PFU/ml), VACV (8 × 10^4^ PFU/ml) and MVM (8 × 10^4^ TCID50/ml) in 0, 1, 10, and 50 μg/ml Echinaforce in cell culture media for 60 min at RT on a rocking platform. 450uL of SARS-CoV-2 (8.33 × 10^4^ TCID_50_/ml) were incubated in the same concentrations of Echinaforce for 60 min at 37 °C on a rocking platform. Residual infectivity was determined by standard plaque assay on Vero cells (MERS-CoV, SARS-CoV-1, YFV and VACV) or a limiting dilution assay on A9 cells (MVM) or Vero E6 cells (SARS-CoV-2) as described below.

### Virus quantification

#### Fifty-percent tissue culture infectious dose (TCID_50_) assay

TCID_50_ for HCoV-229E, MVM and SARS-CoV-2 was determined by limiting dilution assay*.* Briefly, samples were serially diluted 1:10 in 2% FBS - MEM. From each dilution, 100 μl were applied to 10 separate wells of a 96-well plate containing 80% confluent Huh-7, A9 or Vero E6 cells for HCoV-229E, MVM and SARS-CoV-2, respectively. After 7 days of incubation at 33 °C (HCoV-229E), 13 days at 37 °C (MVM) or 3 days at 37 °C (SARS-CoV-2) plates were stained with crystal violet for 15 min (1% aqueous solution, Merck, Zug, Switzerland) and TCID_50_ calculated using the Spearman-Karber Method [27].

#### Plaque assay

Plaque forming units (PFU) for MERS-CoV, SARS-CoV-1, YFV and VACV were determined by standard plaque assay. Briefly, serially diluted samples were titrated on confluent Vero cells in 24-well plates, overlaid with 2% FBS - MEM containing 1.2% methylcellulose (90HG 4000 cP, Sigma Aldrich, Switzerland) and incubated at 37 °C until plaques were clearly visible by microscopy, ranging from 3 to 5 days. For visualization, plates were stained with Crystal Violet (1% aqueous solution, Merck, Zug, Switzerland) for 15 min.

### Statistical analysis

Data was analyzed with one- or two-way ANOVA with a Tukey’s test for multiple comparisons. *P* < 0.05 is considered statistically significant. All analyses were performed with GraphPad Prism, version 8.

## Results

### Echinaforce reduces the infectivity of HCoV-229E in a dose - dependent manner

**Fig. 1:**
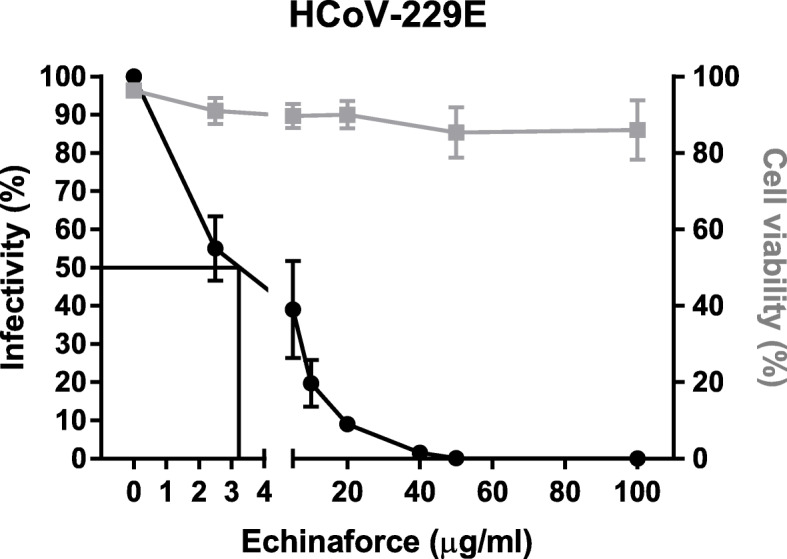
Dose-dependent inactivation of HCoV-229E by Echinaforce. Direct exposure to Echinaforce lead to a dose-dependent inactivation of HCoV-229E. Half-maximal inhibitory concentration, IC_50_, was calculated as 3.2 μg/ml and complete virus inactivation was achieved at a concentration of 50 μg/ml, while no effect was observed on cell viability (right y-axis). The data shown are representative of three independent experiments (mean ± sd)

To assess the direct virucidal activity of Echinaforce against human coronavirus 229E, we exposed 4 × 10^4^ TCID_50_/ml to increasing concentrations of extract and determined the effect on virus infectivity by a limiting dilution assay. Exposure to Echinaforce for 60 min led to a dose - dependent reduction of HCoV-229E infectivity (Fig. 1). Complete inhibition of replicating virus was observed at 50–100 μg/ml extract*;* with half-maximal inhibitory concentration (IC_50_) at 3.2 μg/ml, while parallel incubation of cells with Echinaforce showed stable cell viability at all tested concentrations (Fig. 1).

### Echinaforce affects infectivity through irreversible interactions with HCoV-229E

**Fig. 2:**
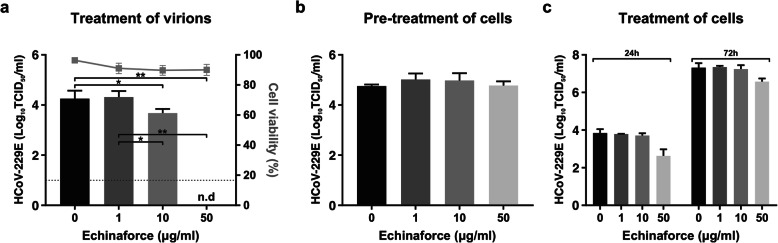
Treatment of cells with Echinaforce does not inhibit HCoV-229E replication. **a** Direct exposure of HCoV-229E to the extract led to a permanent inactivation that could not be reverted by extensive washing. *p = 0.0129, **p = 0.0095. **b** Three day pre-treatment of Huh-7 cells with Echinaforce does not inhibit virus replication. **c** Treatment of Huh-7 cells one-hour post infection (hpi) only resulted in lower viral titers at the highest concentration (50 μg/ml). Dashed line: detection limit, 10 TCID_50_/ml, n.d: not detected at detection limit. The data shown are representative of three independent experiments (mean ± sd)

Since little is known about the mode of action of *Echinacea* extracts we aimed to determine whether Echinaforce exerts its antiviral activity exclusively through direct interaction with virions or also intracellularly during virus replication. To this end, Echinaforce was introduced at different stages of HCoV-229E infection. First, HCoV-229E virus particles were pre-treated prior to infection. Second, cells were treated for 3 days prior to infection. Third, Echinaforce was added to cells one hour post-infection (hpi). Results show, that upon contact with the extract*,* a permanent reduction of virus infectivity occurred, as this virucidal effect could not be reversed through extensive washing of treated virus (Fig. 2a). In contrast, pre-treatment of cells had no influence on HCoV-229E infectivity or replication (Fig. 2b). In cells treated post-infection, a small reduction in virus titer was observed with the highest dose of 50 μg/ml (Fig. 2c).

### Echinaforce inhibits HCoV-229E infection of respiratory epithelial cells

**Fig. 3:**
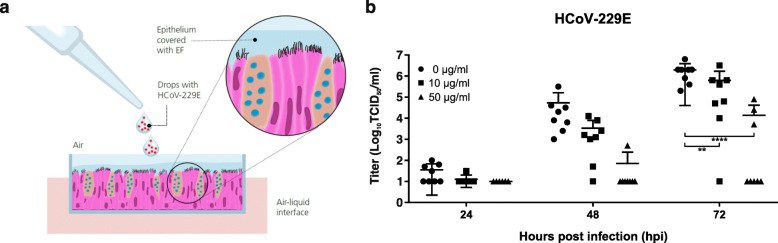
Echinaforce inhibits infection of HCoV-229E in organotypic airway cultures. **a** To simulate natural infection, organotypic nasal epithelial cultures were infected with droplets of HCoV-229E from the apical side. **b** Viral titer in apical secretions was determined at 24, 48 and 72 hpi. Apical pre-treatment with 50 μg/ml lead to complete inhibition of virus replication in 5 out of 8 cultures at 72 hpi, while 10 μg/ml showed complete inhibition only in 1 out of 8 cultures. For both treatment concentrations, a reduction of mean titer was observed when compared to non-treated controls. **p = 0.0015, ****p < 0.0001

To evaluate how Echinaforce may exert its antiviral activity in a more natural setting, we utilized a re-differentiated, pseudostratified respiratory epithelial cell culture model. The reconstituted epithelium is functional, produces mucus and exhibits active ciliary-beating and mucociliary clearance much like in vivo epithelium. To simulate daily usage of the extract, cultures were pre-treated apically with 0, 10, and 50 μg/ml Echinaforce for one day. Subsequently, virus suspension, containing 100 TCID_50_ HCoV-229E was applied dropwise onto the apical surface of the epithelium, simulating common cold exposure and transmission (Fig. 3a). Virus replication was evaluated at 24, 48, and 72 hpi by quantifying infectious virus in apical secretions. In non-treated respiratory epithelium (0 μg/ml), HCoV-229E replicated efficiently. Virus growth could be observed as early as 24 h after infection and virus titers increased over 72 h to a mean of 2 × 10^6^ TCID_50_/ml. In respiratory epithelium pre-treated with 50 μg/ml Echinaforce*,* viral titers remained below detection level in 7 out of 8 cultures at 48 hpi and 5 out of 8 cultures at 72 hpi (Fig. 3b). When virus was not completely neutralized (3/8), the increase of viral titer started later and eventually reached titers that remained 2–3 logs below controls at 72 hpi, indicating a protective effect in the absence of total inactivation (*p* < 0.0001). Since Echinaforce is present on the apical side of the epithelium, this effect is likely due to inactivation of inoculum and/or progeny virus resulting in none or delayed infection. Pre-treatment of respiratory epithelium with 10 μg/ml Echinaforce was less effective; it did nonetheless result in delayed virus growth and reduced viral titers compared to non/treated controls (*p* = 0.002), but completely inhibited virus growth in only 1 out of 8 cultures.

### Echinaforce exhibits virucidal activity against enveloped RNA viruses, including SARS-CoV-1 and SARS-CoV-2

**Fig. 4:**
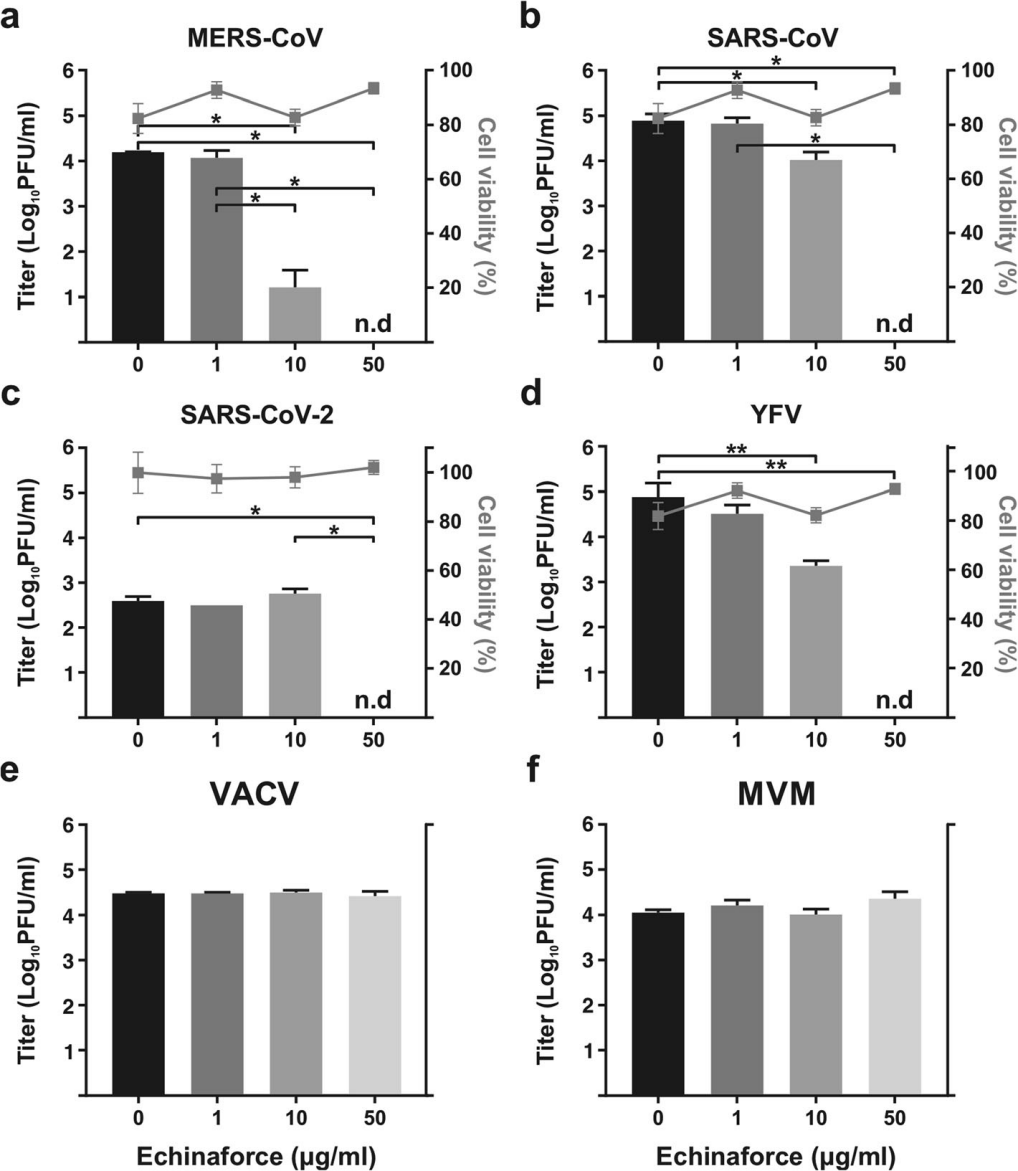
Enveloped RNA viruses are inactivated by direct treatment with Echinaforce. **a** MERS-CoV is highly sensitive to direct Echinaforce treatment, with significant reduction in viral titer observed at 10 μg/ml and complete inactivation at 50 μg/ml. *p = 0.0144 (compared to 0 μg/ml) or *p* = 0.0394 (compared to 1 μg/ml) (**b**) SARS-CoV-1 is completely inactivated at the highest concentration with a slight but significant reduction in viral titer after exposure to 10 μg/ml. *p < 0.0001. **c** SARS-CoV-2 was also completely inactivated after treatment with 50 μg/ml. *p = 0.0452. **d** Exposure to 50 μg/ml Echinaforce leads to complete inactivation of yellow fever virus (YFV) *p = 0.0067. **e** Vaccinia virus and (**f**) mouse parvovirus (MVM) were not sensitive to Echinaforce. No effect was observed on cell viability (right y-axis, (**a**), (**b**), (**c**) and (**d**)). Data shown are representative of two independent experiments (mean ± sd)

Since *Echinacea* preparations have shown a virucidal and antiviral effect against HCoV-229E and other enveloped respiratory viruses [13, 28], we expected to see a similar effect on the related, highly pathogenic coronaviruses MERS-CoV and SARS-CoV-1. To this end, we evaluated the virucidal activity of Echinaforce against these viruses. The observed effects against MERS-CoV (Fig. 4a) and SARS-CoV-1 (Fig. 4b) were comparable with the effects observed for HCoV-229E, with complete inactivation after treatment with 50 μg/ml. Interestingly, MERS-CoV was even more sensitive than HCoV-229E to treatment with a lower concentration (10 μg/ml) of Echinaforce (*p* = 0.0144). Inactivation of the newly identified SARS-CoV-2 was similar to SARS-CoV-1 with complete inactivation at 50μg/ml (Fig. 4c, *p* = 0.0452). Similar virucidal activity was observed for Yellow fever virus (YFV), another enveloped RNA virus (Fig. 4d). In contrast, Echinaforce showed no effect on the infectivity of Vaccinia virus (VACV, Fig. 4e) and the Minute virus of mice (MVM, Fig. 4f), which are DNA viruses, with and without an envelope, respectively.

## Discussion

Broad antiviral therapeutics are of great interest to medicine, as drugs with too high of a specificity rely on quick and accurate pathogen identification and may fail to target genetic variants or newly emerging viruses [29]. Due to the sheer number of different viruses capable of causing respiratory disease and the speed at which symptoms can develop, readily available and broadly effective therapeutics would be highly desirable for both prophylaxis and treatment of respiratory infections. However, for most respiratory viruses, no specific antiviral therapy is available [30–32]. Effective broad-spectrum antivirals would reduce the severity of illness and reduce transmission, thereby lessening the general burden and morbidity of these viruses [33]. Given their penchant for zoonotic transmission, antiviral treatments against highly pathogenic coronaviruses are of particular interest and the current SARS-CoV-2 outbreak further illustrates the need for accessible, fast-acting anti virals.

Herbal preparations of *Echinacea* have traditionally been used to prevent and treat symptoms of colds and flu and are still widely used [10, 13]. Echinaforce, an *Echinacea purpurea* extract, has been shown to broadly inhibit the infectivity of influenza A and B, RSV, parainfluenza virus, and herpes simplex virus in-vitro and to interfere with cytokine production induced upon viral infection [20–22]. Results from the current study complement these previous findings by demonstrating a direct antiviral activity of Echinaforce both against common cold coronavirus 229E (HCoV-229E) and highly pathogenic coronaviruses (SARS-CoV-1 and MERS-CoV). We observed a dose -dependent inactivation of HCoV-229E upon direct exposure to the extract and 50% reduction of HCoV-229E infectivity (IC_50_) was achieved at 3.2 μg/ml. As previously seen for RSV, no intracellular effect was observed for HCoV-229E, as virus replication was not affected by the addition of Echinaforce prior to infection, further suggesting that direct virus contact is required for virucidal activity. This observation, along with the observation that treatment of cell cultures with the extract post infection has only a limited effect at the highest concentration (50 μg/ml), suggests that the observed effects against coronaviruses are restricted to the extracellular phases, i.e. prior to viral entry into the cell and/or during progeny virus release. Furthermore, this virucidal activity is not strain-specific since the related coronaviruses SARS-CoV -1 and − 2, as well as MERS-CoV were inactivated in a comparable manner. Interestingly, even unrelated enveloped RNA viruses such as yellow fever virus were sensitive to Echinaforce treatment indicating a broad antiviral activity against enveloped viruses.

Mechanism of action of different *Echinacea* extracts are currently unclear, however, for most viruses, Echinaforce seems to exert its effect upon direct contact, leading to a permanent inactivation of the virions. In the current study, inhibition of HCoV-229E infectivity after direct exposure could not be reverted by washing. This observed effect is likely due to a stable alteration of exposed viral components, presumably, the viral envelope itself or structural proteins, i.e. the spike glycoprotein (S) or the membrane protein (M). Although specific inhibition has been suggested for Influenza [20], the heterogeneity of the envelope proteins and cell receptors used by all the different viruses susceptible to *Echinacea* treatment strongly argues against a specific mechanism of action. Rather, the broad antiviral activity of *Echinacea* on various enveloped RNA viruses points to a more general inhibitory effect. Non-enveloped rhinoviruses are sensitive to Echinaforce at high concentrations while adenoviruses and mouse parvovirus are not [21]. Interestingly, Echinacea does not inhibit vaccinia virus, a large, enveloped DNA virus. So far, it is the only enveloped virus found to be resistant to treatment with Echinaforce.

We investigated whether a protective effect in the upper-respiratory tract could be reproduced in-vitro, in re-constituted three-dimensional nasal epithelium, i.e. air-liquid interface (ALI) cell cultures, where the apical side is exposed to air resembling the human airways in-vivo. This cell culture system recapitulates many of the characteristics of the human respiratory tract, including ciliary beating and mucus production [34, 35]. Regular intake of Echinaforce was simulated by overlaying cells with a thin layer of the extract and this treatment was sufficient to either prevent or reduce infection with HCoV-229E in respiratory epithelium. Protection against infection with HCoV-229E was observed in 5 out of 8 respiratory epithelial cultures treated with 50 μg/ml after 72 h. At a lower concentration (10 μg/ml), complete protection was only observed in one out of 8 cultures. These results are in agreement with observations made in clinical studies investigating the effect of Echinaforce on the incidence of respiratory tract infections in 755 volunteers. In this randomized, double blind, placebo controlled, clinical study the numbers of cold episodes were significantly lower in the volunteers receiving Echinaforce. While the placebo group had 188 cold episodes, with a collective duration of 850 days, the Echinaforce-treated group had 149 with a duration of 672 days. Throughout the whole study period, 54 viral infections, of which 21 were caused by three of the four common cold coronaviruses, 9 by HCoV-229E, 11 by HCoV-HKU1 and 1 by HCoV-OC43, were detected in the treated group. In contrast, 74 virus infections, of which 33 were coronaviruses, 15: caused by HCoV-229E, 17 by HCoV-HKU1 and 1 by HCoV-OC43) were detected in the placebo group. The same study found that overall infection rates of enveloped respiratory viruses (including HCoV-229E, HCoV-NL63 and HCoV-OC43) were significantly reduced in adults by approximately 50% (*p* = 0.0114) during a 4-month prophylactic treatment with Echinaforce [16]. Furthermore, similar results were recently obtained in a pediatric study where a similar reduction in infection rates was observed in 203 children, aged 4–12 years (*p* = 0.0218) after Echinaforce treatment (Ogal M, unpublished data).

These studies indicate a clinically relevant protection against coronaviruses with prophylactic Echinaforce treatment at tolerable and safe dosages. Furthermore, in the current study we have also observed protection at concentrations lower than required for complete inactivation, indicating that Echinaforce could be beneficial even at suboptimal concentrations. In vivo, this might be due to insufficient dosage or sporadic intake. Better protection may be achieved by ingesting higher doses of the extract or a more directed distribution of Echinaforce in the airways, e.g. by aerosol delivery. Furthermore, isolation and concentration of the active compounds in *Echinacea* products could result in smaller daily doses and increased activity. However, any changes to the current formulations *Echinacea* extracts would require extensive studies into the pharmacokinetics and toxicity of a more concentrated compound.

As previously mentioned, in addition to direct inactivation of viral particles, *Echinacea* also inhibits cytokine secretions during virus infection [36]. Excessive production of interleukin-6 (IL-6) or IL-8 have been associated with symptomatic development of viral infections and such responses, i.e. a cytokine storm, are likely responsible for many of cold-associated symptoms such as runny nose, coughing, sneezing et cetera [37]. During certain viral infections, the heightened immune response may actually contribute to the destruction of respiratory epithelium and may even be the dominant reason for symptoms in absence of virus-mediated cytopathicity [38, 39]. In these cases, the inhibition of virus-induced cytokine production by Echinaforce may be beneficial by limiting the damage of the respiratory epithelium provoked by the immune system [14]. In general, coronaviruses are equipped with various mechanisms to efficiently evade the host immune system and, as a result, do not elicit a pronounced cytokine response early in infection [40]. In accordance, despite severe symptoms and pulmonary pathology, the highly pathogenic MERS-CoV does not elicit an overwhelming cytokine response in primary respiratory epithelial cells in the early course of infection. However, later on, a marked induction of the pro-inflammatory cytokines IL-6 and IL-1β as well as the chemokine IL-8 was observed [41]. This could indicate that the anti-inflammatory action of Echinaforce might be less relevant for coronaviruses, especially the milder ones. However, since treatment with 50 μg/ml Echinaforce inactivated MERS-CoV, SARS-CoV-1 and SARS-CoV-2, the virucidal activity of Echinaforce could still contribute to reduced transmission and milder infections due to the presence of less infectious virus in the upper respiratory tract.

## Conclusions

In the current study, we have shown that four human coronaviruses (HCoV-229E, MERS-CoV, SARS-CoV-1 and SARS-CoV-2) are inactivated by Echinaforce in vitro, further strengthening its use as a prophylactic treatment against a wide range of respiratory viruses causing either serious pulmonary disease or the common cold. Furthermore, a broadly acting antiviral compound suitable for long-term prophylaxis upon exposure could be beneficial to health care workers treating severe CoV infections and potentially reduce the transmission and morbidity of highly pathogenic coronaviruses in the general population. Due to its general mode of action, novel zoonotic coronaviruses, as shown for SARS-CoV-2, could also be sensitive to Echinaforce, potentially providing an accessible and inexpensive prophylactic treatment for other emerging coronavirus infections.

## Data Availability

All relevant source data is available from the corresponding authors upon request.

## Abbreviations

ALI: Air-liquid Interface
ATCC: American Type Culture Collection
CO_2_: Carbon dioxide
CoV: Coronavirus
DER: Drug-to-extract ratio
DMEM: Dulbecco’s Modified Eagle’s Medium
DNA: Deoxyribonucleic Acid
FBS: Fetal Bovine Serum
GMP: Good Manufacturing Practices
HBSS: Hank’s Balanced Salt Solution
HCl: Hydrochloric Acid
HCoV: Human Coronavirus
hpi: Hours post Infection
IC_50_: Half-Maximal Inhibitory Concentration
IL: Interleukin
MEM: Minimum Essential Medium
MERS: Middle East Respiratory Syndrome
MVM: Minute Virus of Mice
PFU: Plaque Forming Units
RNA: Ribonucleic Acid
RSV: Respiratory Syncytial Virus
SARS: Severe Acute Respiratory Syndrome
SDS: Sodium Dodecyl Sulfate
TCID_50_: Median Tissue Culture Infectious Dose
URTI: Upper Respiratory Tract Infection
VACV: Vaccinia Virus
YFV: Yellow Fever Virus
