# Author Correction: In vitro virucidal activity of Echinaforce^®^, an *Echinacea purpurea* preparation, against coronaviruses, including common cold coronavirus 229E and SARS-CoV-2

**DOI:** 10.1186/s12985-020-01439-2

**Published:** 2020-11-09

**Authors:** Johanna Signer, Hulda R. Jonsdottir, Werner C. Albrich, Marc Strasser, Roland Züst, Sarah Ryter, Rahel Ackermann-Gäumann, Nicole Lenz, Denise Siegrist, Andreas Suter, Roland Schoop, Olivier B. Engler

**Affiliations:** 1 SPIEZ LABORATORY, Austrasse, 3700 Spiez, Switzerland; 2 Division of Infectious Diseases and Hospital Epidemiology, Kantonsspital St. Gallen, St. Gallen, Switzerland; 3 A.Vogel AG, Roggwil, Switzerland

## Correction to: Virology Journal (2020) 17:136 10.1186/s12985-020-01401-2

Following publication of the original article [1], the authors would like to make some clarifications:

## Clarification

In the publication *"In vitro virucidal activity of Echinaforce®, an Echinacea purpurea preparation, against coronaviruses, including common cold coronavirus 229E and SARS-CoV-2"*, we describe the virucidal activity of a commercially available formulation, Echinaforce®, against coronaviruses. Our aim was to evaluate the antiviral activity of the product as a whole, rather than specifically investigating the properties of individual components. To this end, we diluted Echinaforce® in cell culture media, 320 times (50 µg/mL) and 1600 times (10 µg/mL). Since our goal was to evaluate the whole extract, we assessed the appropriate negative control as cell culture media alone, rather than the ethanol extraction media used for the Echinacea extraction and production of the product. The concentration of ethanol in the Echinaforce® extract is 65%. However, in our final treatment dilutions, residual ethanol concentrations are 0.2% and 0.04% for 50 µg/mL and 10 µg/mL, respectively. Inactivation of SARS-CoV-2 and other coronaviruses with alcohol-based disinfectants has been shown to require higher concentrations (≥30% v/v) (1, reviewed in 2).

Therefore, in our discussion, we hypothesize that this observed virucidal effect could be due to Echinacea as it has been shown to affect the infectivity of other viruses, when dissolved in either water or ethanol (3). The authors would like to emphasize that direct contact with virus particles is required for virucidal activity and due to the oral administration of Echinaforce® it is currently unclear how relevant this is for *in-vivo* situations. Data on the potential benefits of regular intake of Echinacea for respiratory tract infections is available (4, 5, 6, reviewed in 7) but further research is needed. The authors would like to emphasize that any hypotheses made about the effectiveness of Echinaforce® against SARS-CoV-2 *in-vivo* are theoretical and would need to be investigated in clinical studies (reviewed in 8).

In the current study, we state that the product Echinaforce® - as is – exhibits virucidal activity against four coronaviruses *in-vitro*, upon direct contact in suspension.

Due to concerns regarding the residual ethanol concentrations in our treatment dilutions, we have provided additional data showing no statistical difference in virus replication of viruses previously shown to be sensitive to Echinaforce^®^ between cell culture media and media containing the residual ethanol concentration in our highest treatment dilution (0.2% - “Media + EtOH”) (Fig. 1).

**Fig. 1:**
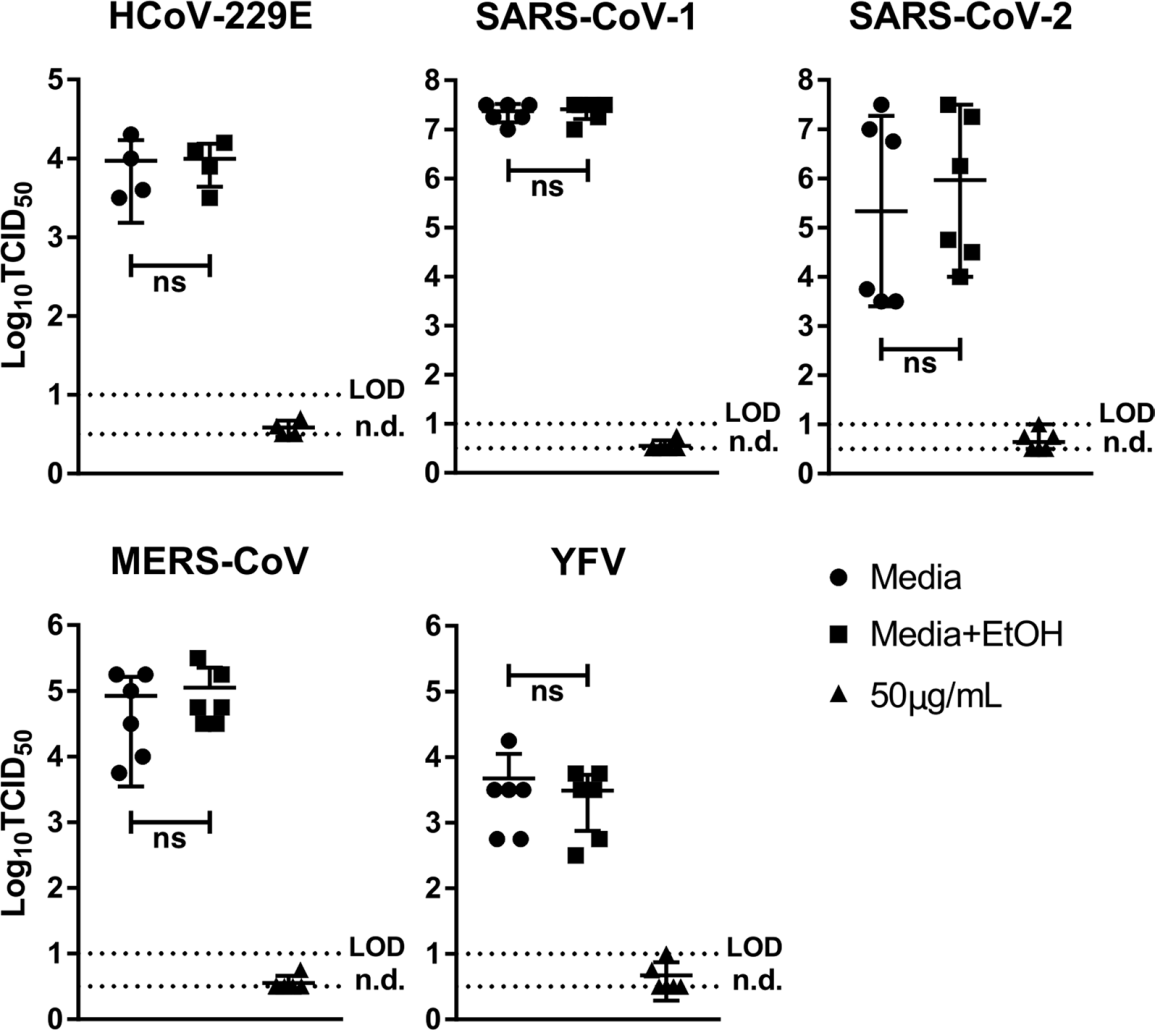
*No virucidal activity against four human coronaviruses and Yellow Fever Virus is observed after direct contact with 0.2% ethanol in suspension.* No significant difference in viral replication of viruses previously shown to be sensitive to Ecinaforce^®^ was observed after treatment with 0.2% ethanol, the residual ethanol concentration corresponding to treatment with 50 µg/mL Echinaforce^®^. These data are generated from two independent experiments in triplicate or duplicate (HCoV-229E) and reported as mean ± sd or geometric mean ± geometric sd (SARS-CoV-2). LOD: limit of detection (10 TCID_50_), n.d: not detected (assigned value: 3.16 TCID_50_/mL)

## Methods

### Echinaforce^®^ treatment

1 × 10^4^ TCID_50_/ml HCoV-229E and 1 × 10^5^ PFU/ml MERS-CoV, SARS-CoV-1 and -2 and YFV were incubated with Echinaforce diluted to 50 μg/ml in 2%-FBS-DMEM (HCoV-229E) or 2%-FBS-MEM and incubated for 1 h at room temperature (RT) on a rocking platform. Cell culture media alone and media containing the corresponding residual ethanol concentration (0.2%) was incubated in the same way.

### Limiting dilution assay (TCID_50_)

Residual infectivity of HCoV-229E, SARS-CoV-1 and -2, MERS-CoV and YFV was determined by a limiting dilution assay (TCID_50_) on Huh7 (HCoV-229E), Vero (YFV) or Vero E6 (SARS-CoV-1 and -2, MERS-CoV) cells according to the Spearman-Kärber algorithm as described by Hierholzer and Killington (1996).

### Statistical analysis

To determine statistical significance, Kruskal-Wallis non-parametric test with Dunn's multiple comparisons test was applied to all data sets using GraphPad Prism version 7.05. P-values < 0.05 were considered statistically significant.

Conflict of interests:

W.C. Albrich wishes to clarify his conflict of interest statement as follows:

While W. C. Albrich has been the recipient of fees and research grants from A. Vogel AG that were paid to his institution, no fees or research grants were received in relation to this article.

References:

1. Kratzel A, Todt D, V'kovski P, et al. Inactivation of Severe Acute Respiratory Syndrome Coronavirus 2 by WHO-Recommended Hand Rub Formulations and Alcohols. *Emerg Infect Dis*. 2020;26(7):1592–1595. doi:10.3201/eid2607.200915
2. Manoj Khokhar, Dipayan Roy, Purvi Purohit, Manu Goyal & Puneet Setia (2020) Viricidal treatments for prevention of coronavirus infection, Pathogens and Global Health, DOI: 10.1080/20477724.2020.1807177
3. Selvarani Vimalanathan, Linda Kang, Virginie Treyvaud Amiguet, John Livesey, J. Thor Arnason & Jim Hudson (2005) *Echinacea purpurea*. Aerial Parts Contain Multiple Antiviral Compounds, Pharmaceutical Biology, 43:9, 740–745, DOI: 10.1080/13880200500406354
4. Barrett B, Brown R, Rakel D, et al. Echinacea for treating the common cold: a randomized trial. *Ann Intern Med*. 2010;153(12):769777. doi:10.7326/0003-4819-153-12-201012210-00003
5. Rauš, Karel et al. “Effect of an Echinacea-Based Hot Drink Versus Oseltamivir in Influenza Treatment: A Randomized, Double-Blind, Double-Dummy, Multicenter, Noninferiority Clinical Trial.” *Current therapeutic research, clinical and experimental* vol. 77 66–72. 20 Apr. 2015, doi:10.1016/j.curtheres.2015.04.001
6. Jawad, M et al. “Safety and Efficacy Profile of Echinacea purpurea to Prevent Common Cold Episodes: A Randomized, Double-Blind, Placebo-Controlled Trial.” *Evidence-based complementary and alternative medicine: eCAM* vol. 2012 (2012): 841315. doi:10.1155/2012/841315
7. Karsch-Völk, M., Barrett, B., & Linde, K. (2015). Echinacea for preventing and treating the common cold. *JAMA*, *313*(6), 618–619. https://doi.org/10.1001/jama.2014.17145
8. Aucoin, Monique et al. “The effect of *Echinacea* spp. on the prevention or treatment of COVID-19 and other respiratory tract infections in humans: A rapid review.” *Advances in integrative medicine*, 10.1016/j.aimed.2020.07.004. 1 Aug. 2020, doi:10.1016/j.aimed.2020.07.004

Due to a typesetting misunderstanding, some cells were merged in the Table 3. The following table displays information correctly. No data have changed from the version originally published.

**Table 3:** Overview of viruses used in the current study

Name	Strain	Propagated in	Medium*	Procured from
HCoV	229E	Huh-7, 33 °C	DMEM + 5%FBS, 2 mM Glutamine, non-essential amino acids, Pen/strep, HEPES (Biochrom, Germany)	Prof. Volker Thiel, University of Bern, Switzerland (24, 25)
MERS-CoV	EMC	Vero, 37 °C	DMEM + 2%FBS, 2 mM Glutamine, non-essential amino acids, Pen/strep, HEPES (Biochrom, Germany)	Prof. Volker Thiel, University of Bern, Switzerland (24, 25)
SARS-CoV	Frankfurt-1	Vero, 37 °C	DMEM + 2%FBS, 2 mM Glutamine, non-essential amino acids, Pen/strep, HEPES (Biochrom, Germany)	Prof. Volker Thiel, University of Bern, Switzerland (24, 25)
SARS-CoV-2	BetaCoV/France/IDF0372/2020	Vero E6, 37 °C	DMEM + 2%FBS, 2 mM Glutamine, non-essential amino acids, Pen/strep, HEPES (Biochrom, Germany)	Institute Pasteur, Paris, France via EVAg, European Virus Archive
Mouse parvovirus	MVM Prototype, ATCC-1346	A9, 37 °C	DMEM + 2%FBS, 2 mM Glutamine, non-essential amino acids, Pen/strep, HEPES (Biochrom, Germany)	The National Collection of Pathogenic Viruses, UK
Yellow Fever virus	17D, NCPV-0507	Vero, 37 °C	DMEM + 2%FBS, 2 mM Glutamine, non-essential amino acids, Pen/strep, HEPES (Biochrom, Germany)	The National Collection of Pathogenic Viruses, UK
Vaccinia virus	Elstree (Lister Vaccine), ATCC-VR-1549	Vero, 37 °C	DMEM + 2%FBS, 2 mM Glutamine, non-essential amino acids, Pen/strep, HEPES (Biochrom, Germany)	The National Collection of Pathogenic Viruses, UK
